# Endoscopy of Nasopharyngeal Cancer

**DOI:** 10.1155/DTE.1.63

**Published:** 1994

**Authors:** Brian B. Burkey, Robert H. Ossoff

**Affiliations:** 1 Vanderbilt University Medical Center S-2100 Medical Center North Nashville Tennessee USA; 2 Department of Otolaryngology Vanderbilt University Medical Center S-2100 Medical Center North Nashville TN 37232-2559 USA

## Abstract

Nasopharyngeal cancer (NPC) is a unique disease with increasing interest for many physicians due to its unusual etiology, histology, and epidemiology. The recent era of fiberoptic endoscopy now provides the clinician with better tools for the screening, diagnosis, staging, and follow-up of NPC. The use of high resolution flexible and rigid nasopharyngoscopy gives the physician an opportunity for a more sensitive examination in a higher proportion of patients. Ultimately, this will allow for earlier diagnosis of NPC, and improved prognosis and better quality of life for the patients with this disease. Also, by allowing the clinician to perform directed biopsies of the nasopharynx under local anesthesia, fiberoptic nasopharyngoscopy allows a less morbid and more cost-effective approach towards this disease, including screening protocols in certain high risk regions of the world.


*Diagnostic and Therapeutic Endoscopy*, Vol. 1, pp. 63–68
Reprints available directly from the publisher
Photocopying permitted by license only

© 1994 Harwood Academic Publishers GmbH
Printed in Malaysia

### Endoscopy of Nasopharyngeal Cancer

BRIAN B. BURKEY and ROBERT H. OSSOFF

*Vanderbilt University Medical Center, S-2100 Medical Center North, Nashville, Tennessee*

*(Received December 16, 1993; in final form January 31, 1994)*

Nasopharyngeal cancer (NPC) is a unique disease with increasing interest for many physicians due to its unusual etiology, histology, and epidemiology. The recent era of fiberoptic endoscopy now provides the clinician with better tools for the screening, diagnosis, staging, and follow-up of NPC. The use of high resolution flexible and rigid nasopharyngoscopy gives the physician an opportunity for a more sensitive examination in a higher proportion of patients. Ultimately, this will allow for earlier diagnosis of NPC, and improved prognosis and better quality of life for the patients with this disease. Also, by allowing the clinician to perform directed biopsies of the nasopharynx under local anesthesia, fiberoptic nasopharyngoscopy allows a less morbid and more cost-effective approach towards this disease, including screening protocols in certain high risk regions of the world.

**KEY WORDS:** anatomy, anesthesia, carcinoma, endoscopy, nasopharynx

### INTRODUCTION

Nasopharyngeal cancer is an uncommon disease in most areas of the world, making up only 0.025% of malignancies in Caucasian males, but it is a disease with enormous interest to many physicians because of its unusual epidemiology, histology, and etiology. (Baker, 1980). The disease occurs throughout life, but has its highest incidence during the fifth and sixth decades. It is rarely seen in North America, with an incidence of 1–2/100,000, but is quite common in the Kwantung province of China, with an incidence as high as 98/100,000, and has an intermediate occurence in areas of North Africa (Cassissi, 1987). Its association with the Epstein-Barr virus, a herpes-group virus, has been firmly established, making it one of a small group of cancers with a strong viral link (Ho *et al.*, 1976). Nasopharyngeal cancer is therefore currently under close study, and of great importance to medical researchers and clinicians alike.

The nasopharynx is lined by mucosa varying from ciliated columnar to stratified squamous epithelium. Carcinomas arising from this surface epithelium therefore can be diverse, but are all included under the heading of nasopharyngeal cancer (NPC). The World Health Organization has divided the tumors into three categories based on histology: types I, II, and III. Type I is a keratinizing squamous cell carcinoma similar to other squamous cell carcinomas of the upper aerodigestive tract. It comprises roughly 25% of all NPC, and has a low incidence in the Chinese population and a weak link to the Epstein-Barr virus (EBV). Type II is a nonkeratinizing carcinoma that accounts for about 15% of all NPC, and is sometimes called transitional carcinoma. Type III, or undifferentiated carcinoma, makes up over 60% of NPC and includes lymphoepithelioma, anaplastic carcinoma, and others. Types II and III together make up a neoplastic process unique to the nasopharynx, and have a strong relationship to the EBV (Weiland, 1987) They can account for 99% of NPC in some Chinese populations.

The etiology of NPC is fiercely debated and is almost certainly multifactorial in nature. Dietary factors are implicated, including an increased intake of nitrosamines, such as in smoked fish, and a decreased intake of vitamin C. As mentioned, the role of EBV in the disease is firmly established, but the exact mechanism has yet to be elucidated. Tumor specimens have been found to contain EBV DNA, and titers of the IgA antibody to the viral capsid antigen (VCA) of EBV increase with the clinical stage of the disease. In fact, the high titers of IgA-VCA found in patients with NPC have allowed researchers in southeastern China to use IgA-VCA titers as a screening marker for the disease (Henle and Henle, 1985)

Address for correspondence: Brian B. Burkey M.D., Assistant Professor, Department of Otolaryngology, Vanderbilt University Medical Center, S-2100 Medical Center North, Nashville, TN 37232-2559.

This is useful because many patients will present with advanced disease, since most symptoms develop only after the tumor has invaded surrounding structures or metastasized. The most common presenting symptom of patients with NPC is that of a neck mass, followed closely by hearing loss from serous otitis media. Nasal complaints including rhinorrhea, congestion, and epistaxis are noted in over one-third of patients. Cranial nerve symptoms are present in 15% of NPC patients, with trigeminal distribution hypesthesia being most common, along with diplopia from abducens nerve dysfunction (Baker, 1981).

Unfortunately, most patients present with advanced disease, that is, stage III or IV NPC. The American Joint Committee for Cancer Staging and End-Results Reporting (AJCC) primary tumor classification and stage grouping classification are listed in Tables 1 and 2 respectively. Five-year survival in this group treated with the standard protocol of radiation therapy to the primary site and necks, followed by salvage neck dissection if necessary, is 20–25%. Overall 5-year survival is 35% (Baker, 1980), but is better in younger patients, those of Chinese descent, and those patients with type III histopathology (Levine *et al.* 1980). The best responses are seen in those patients diagnosed with stage I disease, with up to 70% 5-year survival. Therefore, the need for earlier diagnosis is evident, and this can only be achieved with the use of screening programs in high incidence regions, and improved methods of physical examination.

### ANATOMY

The nasopharynx is a protected area of the upper aerodigestive tract, which is difficult to access for routine examination. This mucosal-lined structure is bounded by the skull base (clivus and sphenoid sinus) superiorly, the cervical vertebrae posteriorly, the posterior choanae of the nasal cavity anteriorly, the superior constrictor muscle laterally, and the isthmus of the soft palate inferiorly. It is adjacent to the paraphayngeal spaces laterally, and this often represents a site of direct tumor extension (Flanders *et al.*, 1991). The nasopharynx contains the torus tubarius, which is the cartilagenous opening of the eustachian tube, and the pharyngeal recess of Rosenmuller, which is just lateral to the torus and a frequent site of development of NPC.(Cassisi, 1987; Sham *et al.*, 1990) Interestingly, the muscular defect at the eustachian tube entrance into the nasopharynx, the sinus of Morgagni, allows tumors in this area to extend into the lateral skull base early in their development.

**Table 1** Primary Tumor (T) Classification for Nasopharyngeal Carcinoma According to the American Joint Committee for Cancer Staging and End-Results Reporting

| *T classification* | *Description* |
|---|---|
| TIS | Carcinoma in situ |
| T1 | Tumor confined to one site of nasopharynx |
| T2 | Tumor involving two sites (both postero-superior and lateral walls) |
| T3 | Extension of tumor into nasal cavity or oropharynx |
| T4 | Tumor invasion of skull or cranial nerve involvement |

**Table 2** Stage Grouping for Nasopharyngeal Carcinoma According to the American Joint Committee for Cancer Staging and End-Results Reporting

| *Stage* | *Grouping* |
|---|---|
| I | T1 NO MO |
| II | T2 NO MO |
| III | T3 NO MO |
| | T1 or T2 or T3, N1 MO |
| IV | T4, NO or N1, MO |
| | Any T, N2 or N3, MO |
| | Any T, any N, M1 |

### ENDOSCOPIC TECHNIQUES

Traditionally, examination of the nasopharynx is performed with a headlight and a postnasal mirror. This indirect nasopharyngoscopy gives an adequate view of the anatomy in most patients. Patients with a narrow nasopharynx, bulky tongue, or redundant soft palate, however, will be difficult to examine by this method. Those with a sensitive gag reflex will make mirror examination of this region impossible (Woo and Sham, 1990) The direct endoscopic techniques allow the clinician a more reliable and higher resolution view of the nasopharynx. Direct nasopharyngoscopy usually requires some type of local anesthesia in order to ensure patient comfort and compliance with the examination. This can be accomplished with the topical application of an anesthestic transnasally. A 5–10% cocaine solution or a 4% lidocaine solution have provided adequate anesthesia for some (Becker *et al.*, 1992; Chiang *et al.*, 1977; Shanmugham, 1985) although the authors have obtained excellent results with a 1% tetracaine with 0.5% phenylephrine solution applied transnasally, supplemented with transoral cetacaine spray when necessary. The transoral anesthesia of the pharynx is usually needed only for rigid nasopharyngoscopy.

Rigid endoscopy of the nasopharynx is an important technique that is simple to perform. A set of Hopkins rod telescopes with 0-degree, 30-degree, and 90-degree lenses is required. The 0-degree and 30-degree telescopes are passed transnasally to evaluate the roof and recesses of the nasopharynx (Figs. 1 and 2). The 90-degree telescope is inserted transorally, with or without retraction of the palate, and provides a wide-angle view of the nasopharynx which is unmatched in clarity and allows detection of minor assymetries in the anatomy. This latter view is quite familiar to the clinician accustomed to indirect nasopharyngoscopy, but affords him a much higher degree of resolution (Figs. 3 and 4). The rigid technique may be particularly useful in

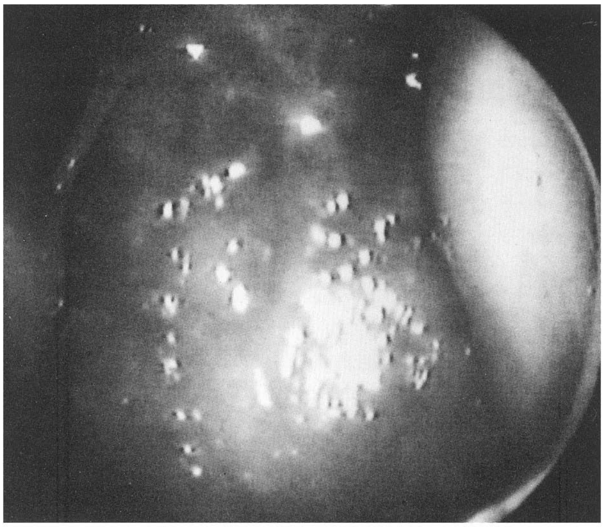

**Figure 1** The nasopharyngeal vault in a normal patient as viewed through a 0-degree rigid nasopharyngoscope in the right posterior choana. The right torus tubarius and the posterosuperior wall are evident, as well as the edge of the left torus tubarius.

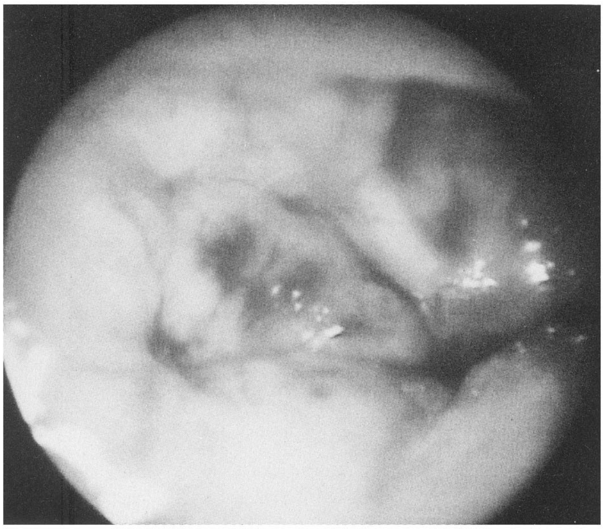

**Figure 2** The posterosuperior wall of the nasopharynx in a 28-year-old female with bilateral serous otitis media. This view was obtained with a 30-degree rigid nasopharyngoscope placed in the right nasal cavity and advanced into the nasopharynx. Biopsy of this lesion revealed a small cell carcinoma.

cases of NPC with small primary lesions, and may provide better photo documentation of disease than flexible endoscopy, when desired (Chiang *et al.*, 1977).

The flexible fiberoptic bronchoscope was introduced in 1970, and was a welcome addition to the armamentarium of the endoscopist. Modifications made since that time have resulted in the modern nasopharyngoscope, which affords excellent visualization of all anatomic areas of the nasopharynx (Silberman *et al.*, 1976). This flexible device is passed transnasally, and has a small diameter so that biopsy forceps can be passed along side of it and biopsies performed under direct visualization, when necessary. With manipulation of the tip of the scope, the entire nasopharynx can be viewed from one nasal cavity. In general, all patients tolerate this method of examination and no complications have been reported (Shanmugham, 1985). Flexible nasopharyngoscopy is particularly useful in those 6% of patients with trismus as a result of local spread of NPC, as it is the only method which allows an adequate exam in this scenario (Chiang *et al.*, 1977). It is also the procedure of choice when a biopsy of a lesion is desired, since it easily allows directed biopsies of suspected NPC with only local anesthesia. Either form of direct endoscopic nasopharyngoscopy provides the clinician the opportunity to record the images obtained from the nasopharynx on videotape, which can then be stored indefinitely, and be used to compare with future images obtained from the same patient or with different images from other patients. Videonasopharyngoscopy requires a nasopharyngoscope, a light source, a video camera, a color monitor, and a VHS recorder/player. A video imager is optional in such a set-up, but allows the added flexibility of making hard copy images of the nasopharynx directly from the video camera, without having to use a separate adapter and 35mm camera. Images recorded with a video system, both on videotape or as a hard copy, can then be stored in a video library and retrieved at any time for comparison with previous exams, or for use in data analysis in clinical investigations.

The light source preferred by the authors for direct endoscopic nasopharyngoscopy is xenon. The xenon lamp has a color temperature of 5500 degrees Kelvin, and provides for excellent color balance with both video imaging and daylight 35mm film. It is a bright source, and easily adapted to both office and operating room settings.

### APPLICATIONS OF NASOPHARYNGOSCOPY

Fortunately, the vast majority of NPC grows in an exophytic fashion, and is simple to diagnose on direct (flexible or rigid) nasopharyngoscopy. In one study utilizing

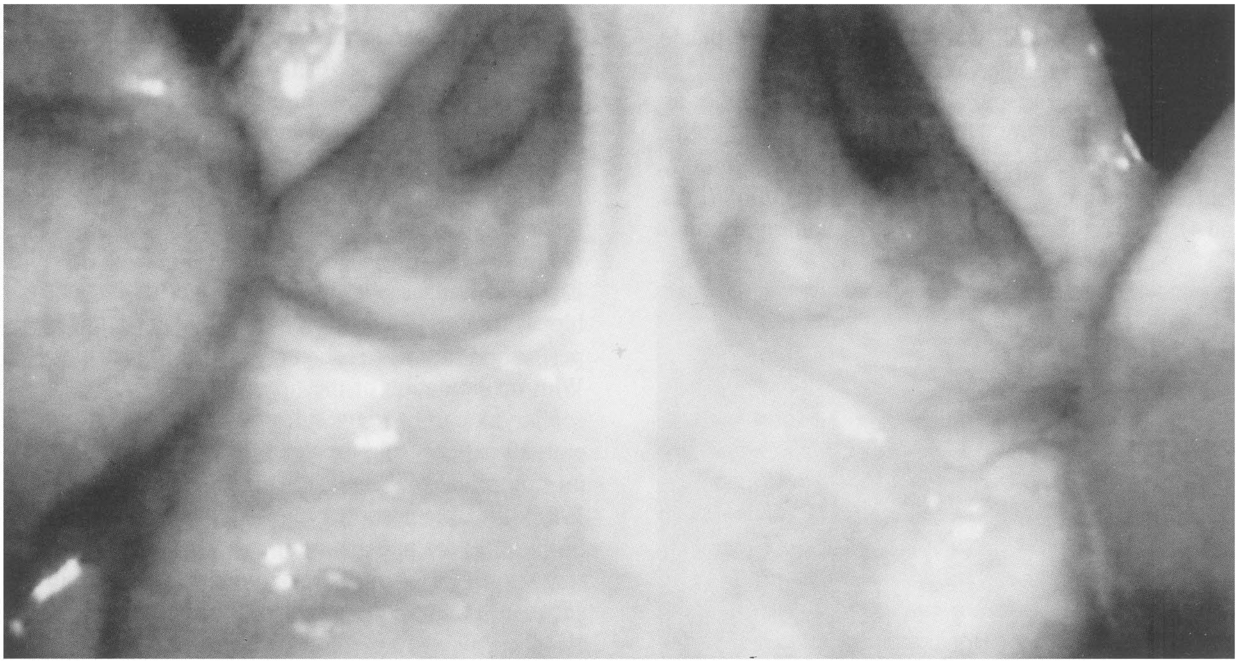

**Figure 3** The entire nasopharynx as viewed through a 90-degree rigid nasopharyngoscope placed transorally. Both eustachian tube orifices, the posterior nasal cavity, and the posterosuperior wall are well defined. The lateral recesses are without tumor in this normal patient.

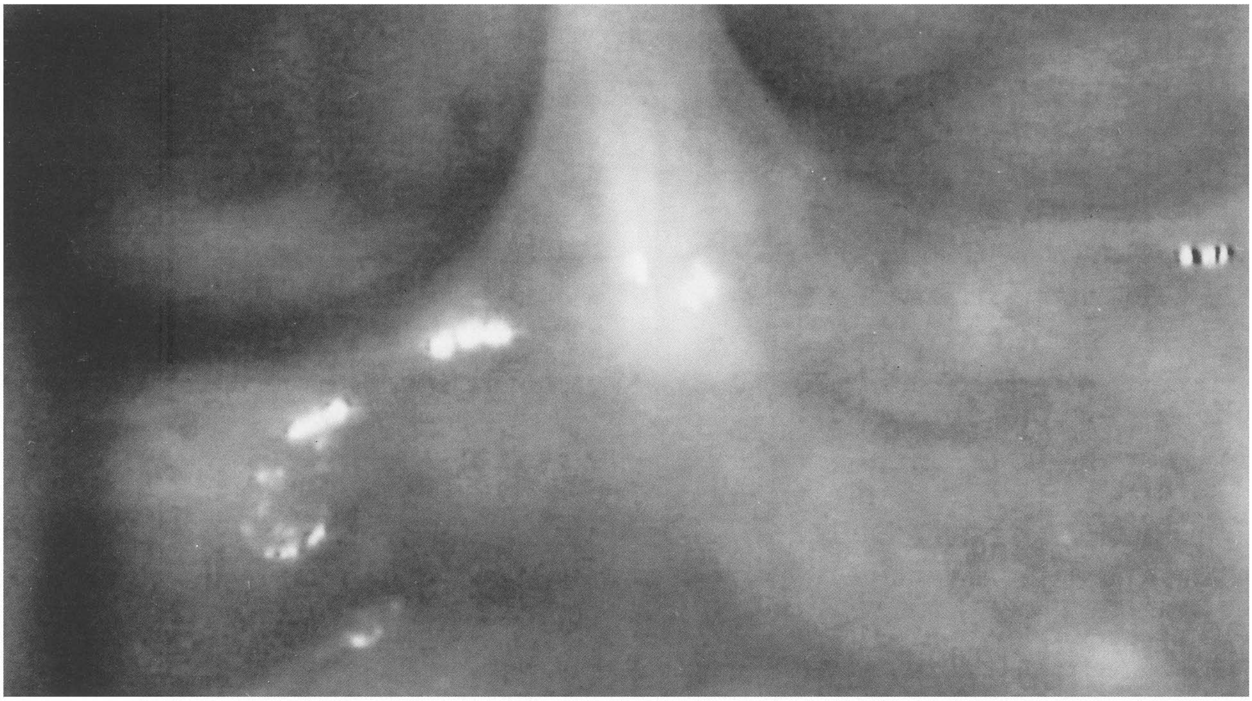

**Figure 4** A close-up view of the vault of the nasopharynx in the same patient as in Figure 3. The superior aspect of the posterior nasal septum is noted in the midline top of the figure.

flexible nasopharyngoscopy, the concordance between visual detection of NPC and biopsy detection of NPC was 73%. The most common reason for not detecting the tumor at a specific site in the nasopharynx was occult spread, which occurred most often on the posterior wall (Sham *et al.*, 1989). One obvious application for direct nasopharyngoscopy, therefore, is the diagnosis of NPC in the high risk patient. The sensitivity of this exam can be increased further by flushing the nasopharynx with 1% hydrogen peroxide prior to endoscopy, then areas of ulceration will degrade the liquid and appear as white patches against the pink mucosal background (Qin *et al.*, 1985).

In southeastern China, where NPC is the most common head and neck tumor, direct nasopharyngoscopy has been combined with the use of serologic screening in trials assessing the early detection of NPC. In one such study, 5.5% of the population of Wuzhou City aged 40 or over was IgA-VCA positive and these patients were followed clinically for four years. Thirty-five cases of NPC were diagnosed in these high risk individuals (3.1%), but 91.5% of cases were detected in either stage I or II, where the cure rates are the highest (Zeng *et al.*, 1985). In fact, the stage of disease at diagnosis is the most important prognostic factor for NPC (Wei *et al.*, 1987).

The benefits of direct nasopharyngoscopy can further be illustrated by another such screening study by Sham *et al.* Over 6,000 IgA-VCA positive individuals were observed, and 130 picked at random and deemed free of disease by mirror exam and random biopsy of the nasopharynx. These latter 130 patients were then examined by flexible nasopharyngoscopy, and biopsies taken under direct visualization from six sites in the nasopharynx. Seven cases of NPC were detected, most in the lateral recess and most with visually defined abnormalities. This represented 5.4% of the study population, while the diagnosis rate of NPC in the IgA-VCA positive population in the same study was only 0.8% by conventional methods of detection (Sham *et al.*, 1990). The addition of flexible nasopharyngoscopy to the study protocol increased the sensitivity of exam both by providing a higher resolution view of the nasopharynx than mirror exam could provide, and by allowing directed biopsies to be taken under simultaneous visualization. All patients were biopsied under local anesthesia alone. Additional uses of nasopharyngoscopy in NPC include the staging of the primary tumor. More sensitive viewing of the tumor can upgrade T1 lesions to T2 lesions, which will not effect therapy, but may aid in reporting results, and determining prognosis. T3 lesions should be detectable on routine mirror examination of the nasopharynx. Obviously, complete staging still requires the use of radiographic procedures, which can show deep extension of disease not appreciable by any method of direct nasopharyngoscopy. The CT scan is best at showing subtle bony changes at the skull base, and MRI scanning is excellent at soft tissue and central nervous system imagery (Braun, 1989). These radiographic techniques play complimentary roles in the staging of NPC.

Finally, follow-up examination after the treatment of NPC is greatly facilitated by either rigid or flexible direct nasopharyngoscopy. Frequent re-examination of the nasopharynx is essential since 50% of all recurrences involve the primary site (Baker, 1980). The increased resolution provided by the fiberoptic scopes gives the clinician a higher degree of certainty in the physical examination (Fig. 5). Videonasopharyngoscopy can also provide for a better comparison between previous examinations, and demonstrate the true evolution of a lesion from diagnosis, through treatment and during serial follow-up. Unnecessary additional studies, such as MRI/CT scans or biopsies, can therefore be avoided. Earlier detection of recurrences can be made as well, and directed biopsies performed under local anesthesia, so that subsequent therapy can be instituted in a timely fashion. Hopefully, earlier detection of recurrences can also lessen the magnitude of salvage surgery when necessary.

### CONCLUSION

Nasopharyngeal cancer is a unique disease with increasing interest for many physicians due to its unusual etiology, histology, and epidemiology. The recent era of fiberoptic endoscopy now provides the clinician with better tools for the screening, diagnosis, staging, and follow-up of NPC. The use of high resolution flexible and rigid nasopharyngoscopy gives the physician an opportunity for a more sensitive examination in a higher proportion of patients. This ultimately will allow for earlier diagnosis of NPC, and improved prognosis and better quality of life for the patients with this disease. Also, by allowing the clinician to perform directed biopsies of the nasopharynx under local anesthesia, fiberoptic nasopharyngoscopy allows a less morbid and more cost-effective approach towards this disease, including screening protocols in certain high risk regions of the world.

The future holds great promise for the study and treatment of NPC, particularly in light of the advances in nasopharyngoscopy. Improved screening techniques are being tested in clinical trials and more aggressive, and successful, salvage surgical procedures are being carried out and refined (Chen *et al.*, 1989) Patient education is also being carried out to help the afflicted individuals overcome the physical and emotional trauma associated with

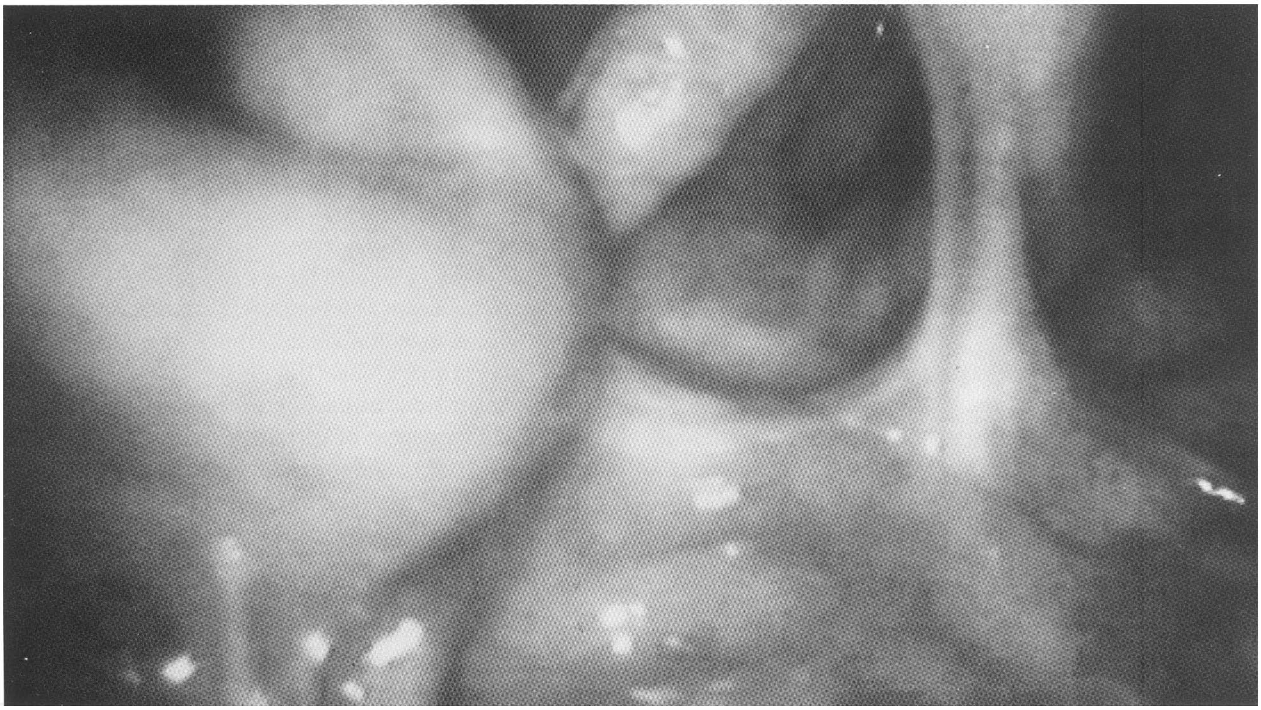

**Figure 5** The right side of the nasopharynx in a 38-year-old male 1-year status post radiation therapy for a T2N2bMO squamous cell carcinoma originating in the right lateral recess. No disease is clinically evident at the primary site, as can be seen in this view obtained with a 90-degree rigid nasopharyngoscope placed transorally. A follow-up MRI was also negative for disease, as was the neck exam.

NPC, and its treatment, and to enable the patient to regain a more productive lifestyle.

### REFERENCES

American Joint Committee for Cancer Staging and End-Results Reporting (1977) Manual for Staging of Cancer, American Joint Committee, Chicago.

Baker S. R. (1981) Malignant tumors of the nasopharynx. *J Sur Oncol* 17:25–32.

Baker S. R. (1980) Nasopharyngeal carcinoma: clinical course and results of therapy. *Head & Neck Surg* SEPT/OCT:8–14.

Becker S. P., Roberts N., Coglianese D. (1992) Endoscopic adenoidectomy for relief of serous otitis media. *Laryngoscope* 102:1379–1384.

Braun I. F. (1989) MRI of the nasopharynx. *Radiologic Clinics of North America* 27:315–330.

Cassisi, N. J.: Clinical evaluation of pharyngeal tumors. In: Thawley S., Panje W. (eds) (1987) *Comprehensive Management of Head and Neck Tumors*, W. B. Saunders Co., Philadelphia.

Chen J. Y., Chen C., Liu M., Cho S., Hsu M., Lynn T., Shieh T., Tu S., Beasley R. P., Hwang L., Lee H., Kuo S., Yang C. (1989) Antibody to Epstein-Barr virus-specific DNase as a marker for field survey of patients with nasopharyngeal carcinoma in Taiwan. Journal of Medical Virology, 27:269–273.

Chiang T. C., Jung P. F. (1977) The nasopharyngoscope and camera examination of the primary carcinoma of nasopharynx. *Cancer* 40:2353–2364.

Flanders A. E., Helinek G. L., Tom B. M., Rao V. M. (1991) Imaging of the nasopharynx. *Crit Rev* Diagn Imaging, 31(3,4):357–411.

Henle W., Henle G. (1985) Epstein-barr virus and human malignancies. In: Klein G. (ed): *Advances in Viral Oncology*, Raven Press Ltd., New York.

Ho H. C., Ng M. H., Kwan H. C., Chau J. C. W. (1976) Epstein-barr-virus-specific IgA and IgG serum antibodies in nasopharyngeal carcinoma. *Brit J Can* 34:655–660.

Levine P. H., Connelly R. R., Easton J. M. (1980) Demographic patterns for nasopharyngeal carcinoma in the United States. *Int J Canc* 26:741–748.

Qin D., Hu Y., Gu X. (1985) Improved technique for the diagnosis of atypical nasopharyngeal cancer. *Laryngoscope*. 95:478–480.

Sham J. S. T., Wei W. I., Kwan W. H., Chan C. W., Choi P. H. K., Choy D. (1989) Fiberoptic endoscopic examination and biopsy in determining the extent of nasopharyngeal carcinoma. *Cancer* 64:1838–1842.

Sham J. S. T., Wei W. I., Yong-Sheng Z., Choy D., Yan-Qin G., Yan L., Zhi-Xiong L., Ng M. H. (1990) Detection of subclinical nasopharyngeal carcinoma by fibreoptic endoscopy and multiple biopsy. *The Lancet*, 335:371–374.

Shanmugham M. S. (1985) The role of fibreoptic nasopharyngoscopy in nasopharyngeal carcinoma (NPC). *J Laryngol Otol* 99: 779–782.

Silberman H., Wilf H., Tucker J. A. (1976) Flexible fiberoptic nasopharyngolaryngoscope. *Ann Otolaryngol* 85:640–645.

Wei W. I., Lau W. F., Lam K. H., Hui Y. (1987) The role of the fibreoptic bronchoscope in otorhinolaryngological practice. *J Laryngol Otol* 101:1263–127.

Weiland L. H. (1987) Pathology of pharyngeal tumors. In: Thawley S., Panje W. (eds): Comprehensive Management of Head and Neck Tumors, W. B. Saunders Co., Philadelphia.

Woo J. K, Sham C. L. (1990) Diagnosis of nasopharyngeal carcinoma. *Ear, Nose and Throat Journal*, 69:241–252.

Zeng Y., Zhang L. G., Wu Y. C., Huang Y. S., Huang N. Q., Li J. Y., Wang Y. B., Jiang M. K., Fang Z., Meng N. N. (1985) Prospective studies on nasopharyngeal carcinoma in Epstein-Barr virus IgA/VCA antibody-positive persons in Wuzhou City, China. *Internat J Cancer*, 36:545–547.

